# Effects of Si and N Addition on *Oryza sativa* and Its Invasive Grazer Apple Snails (Ampullariidae): Resistance Traits and Feeding Metrics

**DOI:** 10.3390/plants15152357

**Published:** 2026-07-30

**Authors:** Wei Li, Jing-Yi Zhao, Hua Yu, Wen-Hong Dai, Yao-Bin Song, Ming Dong

**Affiliations:** Key Laboratory of Hangzhou City for Ecosystem Protection and Restoration, Zhejiang Provincial Key Laboratory of Wetland Intelligent Monitoring and Ecological Restoration, College of Life and Environmental Sciences, Hangzhou Normal University, Hangzhou 311121, China; liwai33442021@163.com (W.L.); 2024111010070@stu.hznu.edu.cn (J.-Y.Z.); huayu@hznu.edu.cn (H.Y.); dwh@hznu.edu.cn (W.-H.D.)

**Keywords:** biological invasion, eutrophication, herbivory, plant defense, silicon addition

## Abstract

Silicon, as a crucial element of plants, contributes to plants’ resistance to biotic stresses like herbivore feeding. In tropical and subtropical Asia, the apple snail (Ampullariidae) is an invasive aquatic herbivorous snail in agricultural and wetland ecosystems, like paddy fields, which suffer from heavy nitrogen eutrophication. However, little is known about the potential effects of supplementing rice leaves treated with silicon and nitrogen on the feeding metrics of apple snails (*Pomacea* spp.). This study conducted a greenhouse experiment to examine the effects of silicon and nitrogen addition on rice seedlings grazed by apple snails, with two levels of silicon addition (0 and 1.5 mM) and three levels of nitrogen addition (0.72, 1.44 and 5.76 mM). The results showed that silicon addition increased plant mass under low and high nitrogen addition, and increased the C/N ratio and tannin content only under the middle nitrogen addition, while it increased the leaf sulfur content of rice at all three levels of nitrogen addition. However, silicon addition significantly decreased the growth of apple snails while significantly increasing flavonoid content and the force of fracture of rice leaves across all nitrogen levels. This study indicates that silicon addition could improve the defense of rice against herbivory by invasive apple snails, providing insights into the protection of wetland crops in the context of eutrophication and biological invasion.

## 1. Introduction

Apple snail (Ampullariidae), native to South America, is invasive in Asia and has been listed among the world’s top 100 worst invasive species [1]. This snail primarily feeds on aquatic plants and crops [2], negatively affects the biomass of wild macrophytes, water quality, and algal production in the invaded range [3,4], and has caused enormous damage to wetland ecosystems and agricultural production, especially in rice-planting areas [5,6,7]. The snail primarily consumes vegetables and aquatic plants, which can lead to a decrease in shoot biomass and vegetation cover in invaded communities [8]. This feeding activity can result in a 50% reduction in agricultural production [9], ultimately diminishing the ecosystem services of wetlands and farmlands. This snail causes significant economic losses in its non-native habitats, including China [10]. Due to the diverse geographical landscapes and varied climatic conditions, China is vulnerable to invasions by the apple snail [11]. In China, the apple snail has invaded the main rice (*Oryza sativa*) planting areas, ranging from the Yangtze River Basin, leading to huge crop production losses [12,13,14]. The snail caused the most damage to 21-day-old transplanted rice seedlings, which can result in up to 100% crop loss [15], and it can constantly damage the rice crop. After harvest, snails can conceal themselves in the mud, posing a threat to the rice crop in the following year [16]. By 2006, the area affected by apple snails in rice fields had exceeded 300,000 hectares, resulting in a direct loss of up to 12.5 million dollars [17]. Unfortunately, massive physical and chemical measures used to eradicate the apple snail in invaded ranges have shown limited efficiency [18,19].

Silicon is recognized as a beneficial element for plants, particularly grasses, not only due to its high dry-weight concentration [20], but also for its vital role in mitigating abiotic and biotic stress [21,22,23]. Silicon can help plants mitigate damage caused by herbivores in multiple ways. Firstly, silicon can enhance plant defense through the deposition of inorganic amorphous silicon oxide (SiO_2_) phytoliths in the epidermis of plant tissues [24]. For example, Johnson et al. found that supplementing plants with silicon resulted in a 50% reduction in the growth rate of an aphid (*Rhopalosiphum padi*) [25]. On the other hand, silicon can also activate the jasmonic acid and salicylic acid metabolic defense pathways [26] that are often associated with leaf damage by herbivores [27]. Moreover, silicon supports the production of defensive secondary metabolites in plants and boosts the activity of oxidation-related enzymes [28], resulting in a toxic impact on herbivores and lowering their preference for these plants. In addition, silicon can improve the release of volatile organic compounds by plants, which can attract the natural enemies of herbivores [29,30] and interfere with the recognition of the host plants by herbivores [31]. Therefore, silicon could help rice alleviate the herbivory stress of invasive apple snail.

Nitrogen is essential for plant growth and reproduction [32], and it plays a critical role in herbivores that is significantly affected by the consumption of high-nitrogen food [33]. Due to overloading of reactive nitrogen from food production and energy production [34], most ecosystems suffer from eutrophication [35]. Jamieson and Bowers found that the growth of *Calophasia lunula* in *Linaria dalmatica* was promoted by nitrogen enrichment, and plants have evolved to balance the trade-offs among growth, reproduction and defense [36,37]. The excess nitrogen deposition may reduce defense costs [35,38], and the increase in nitrogen availability can lead to changes in their secondary metabolites [39], which are associated with plant resistance to herbivores. Moreover, available nitrogen concentration could significantly affect the silicon uptake of plants because silicon is considered a cheap defensive substance [40]. Therefore, a trade-off exists between silicon and nitrogen accumulation in Si-accumulating plant species, potentially having significant implications for their defense mechanisms against invasive herbivores [41]. However, there is no statement on whether there are interactive effects of silicon and nitrogen on the growth of rice and the invasive apple snails.

In this study, we conducted a four-month controlled experiment on rice plants using three levels of nitrogen supply combined with two levels of silicon addition. Subsequently, apple snails were fed with rice leaves from the different treatments for one week in the laboratory to test the following questions: (1) Does silicon addition under different nitrogen levels affect the growth and development of rice? (2) Do different nitrogen levels affect silicon’s ability to defend rice against apple snail when they feed on silicon-treated rice leaves?

## 2. Results

### 2.1. Leaf Resistance Characteristics Under Silicon and Nitrogen Addition

**Figure 1:**
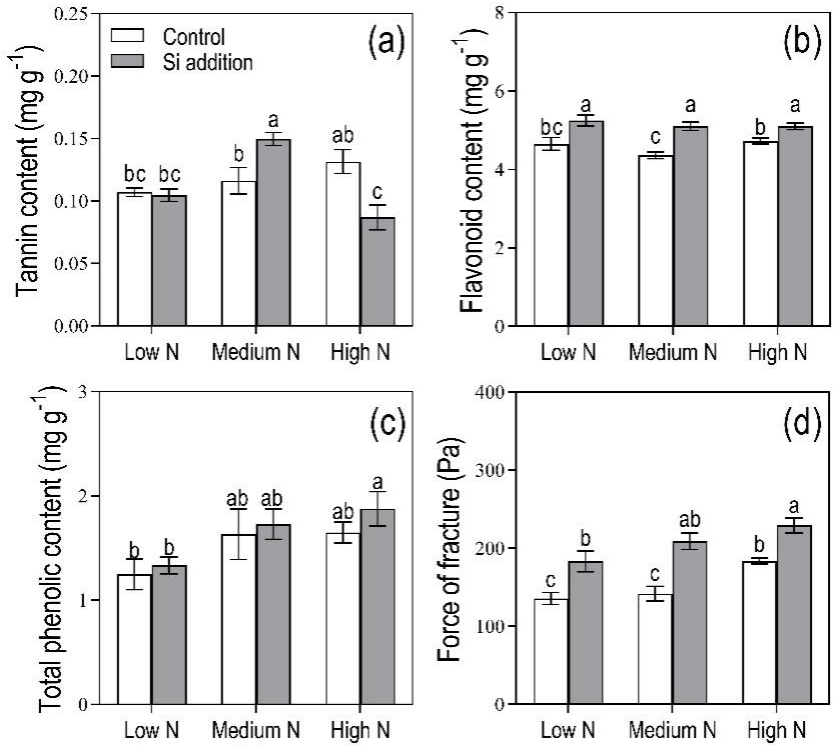
Effects of silicon addition and nitrogen levels on leaf resistance characteristics of rice, including tannin content (**a**), flavonoid content (**b**), total phenolic content (**c**), and fracture force (**d**), with Si addition (gray bar) and without Si addition (white bar), respectively. Values are presented as Means ± SE. Bars with different letters indicate significant difference (*p* < 0.05).

Silicon addition significantly affected flavonoid content (*p* < 0.001) and leaf fracture force (*p* < 0.001), while nitrogen levels significantly affected total phenolic content (*p* = 0.011), tannin content (*p* = 0.003) and leaf force of fracture (*p* < 0.001). A significant interaction was found for tannin content (*p* < 0.001) (Table 1), with silicon increasing tannin content under middle nitrogen levels but decreasing it under high nitrogen levels (Figure 1a). Silicon consistently increased flavonoid content across nitrogen levels, with the minimum under medium nitrogen (Figure 1b), but had no effect on total phenolic content (Figure 1c). Both silicon addition and high nitrogen significantly increased leaf fracture force (Figure 1d), and total phenolic content and leaf fracture force increased with nitrogen level (Figure 1c,d).

### 2.2. Snail Feeding Metrics Under Silicon and Nitrogen Addition

**Figure 2:**
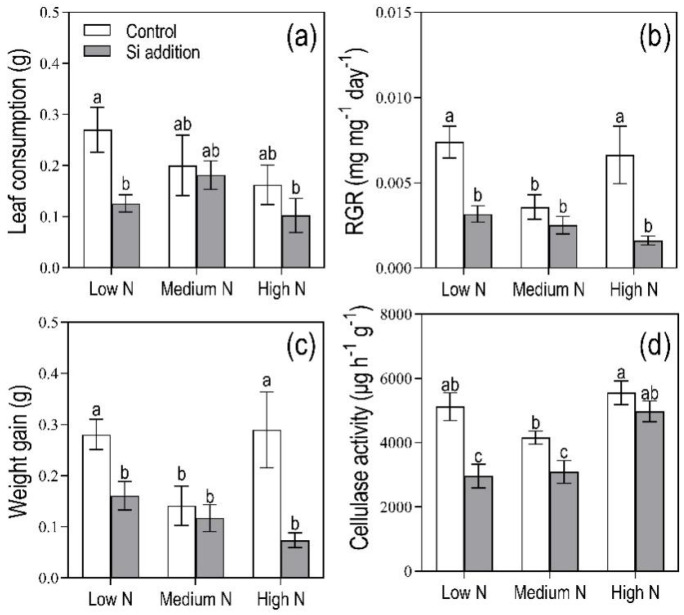
Effects of silicon addition and nitrogen levels on snail feeding metrics, including leaf consumption (**a**), relative growth rate (RGR) (**b**), weight gain (**c**), and cellulase activity (**d**) of apple snails, with Si addition (gray bar) and without Si addition (white bar), respectively. Values are presented as Means ± SE. Bars with different letters indicate significant difference (*p* < 0.05).

Silicon addition significantly affected leaf consumption (*p* = 0.026), relative growth rate (RGR, *p* < 0.001), weight gain (*p* = 0.001), and cellulase activity (*p* < 0.001) of apple snails. Nitrogen level significantly influenced cellulase activity (*p* < 0.001), but did not significantly affect the other feeding metrics. Medium N application alone significantly reduced RGR, weight gain, and cellulase activity. No significant interaction between nitrogen levels and silicon addition was detected for any of the snail feeding metrics (Table 1). Silicon addition significantly reduced leaf consumption, RGR, and weight gain of apple snails under both low and high nitrogen levels (Figure 2a–c). Silicon addition decreased cellulase activity in apple snails, whereas both low and high nitrogen levels increased cellulase activity (Figure 2d).

### 2.3. Leaf Element Contents Under Silicon and Nitrogen Addition

**Figure 3:**
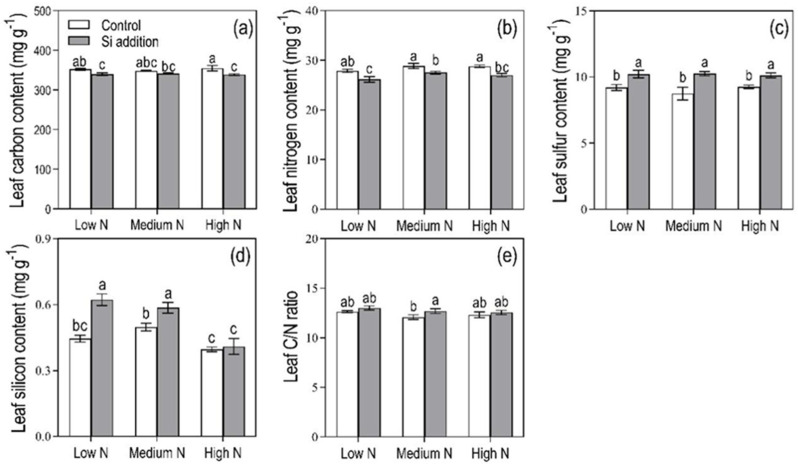
Effects of silicon addition and nitrogen levels on leaf element content of rice leaves, including leaf carbon content (**a**), nitrogen content (**b**), sulfur content (**c**), silicon content (**d**), and C/N ratio (**e**), with Si addition (gray bar) and without Si addition (white bar). Values are presented as Means ± SE. Bars with different letters indicate significant difference (*p* < 0.05).

Silicon addition significantly affected leaf N (*p* < 0.001), C (*p* < 0.001), S (*p* < 0.001), Si (*p* < 0.001), and leaf C/N ratio (*p* = 0.038). Nitrogen level significantly affected leaf N (*p* = 0.020) and leaf Si (*p* < 0.001), with a significant interactive effect between silicon addition and nitrogen level observed only for leaf Si (*p* = 0.006) (Table 1). Silicon addition significantly decreased leaf carbon and nitrogen content across all nitrogen levels (Figure 3a,b), while significantly increasing leaf sulfur content under all nitrogen levels (Figure 3c). Silicon addition significantly increased leaf silicon content under low and medium nitrogen levels, whereas leaf silicon content was lowest under high nitrogen levels (Figure 3d). Silicon addition increased the leaf C/N ratio under medium nitrogen levels (Figure 3e).

### 2.4. Plant Growth Under Silicon and Nitrogen Addition

**Figure 4:**
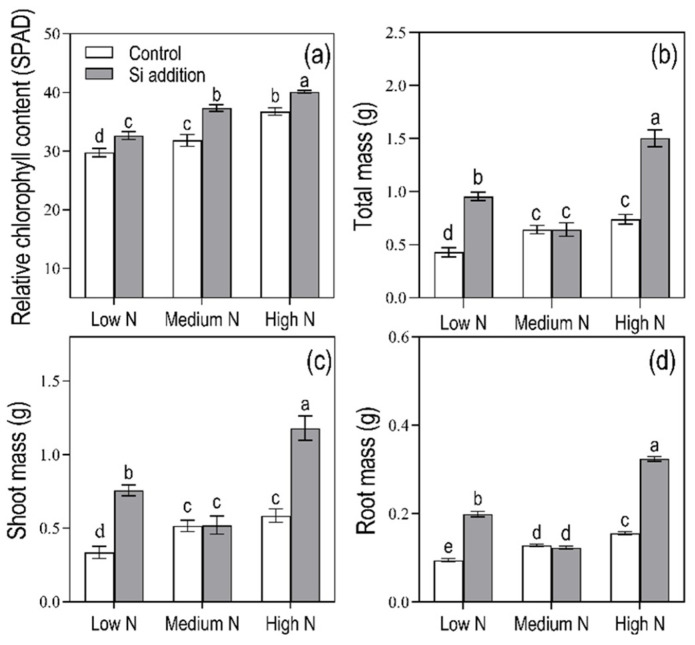
Effects of silicon and nitrogen additions on rice growth, including relative chlorophyll content (**a**), total biomass (**b**), shoot mass (**c**) and root mass (**d**) of rice, with Si addition (gray bar) and without Si addition (white bar), respectively. Values are presented as Means ± SE. Bars with different letters indicate significant difference (*p* < 0.05).

Silicon addition and nitrogen level significantly affected relative chlorophyll content, total mass, shoot mass, and root mass of rice (all *p* < 0.001). Significant interactions between nitrogen level and silicon addition were observed for total mass, shoot mass and root mass (all *p* < 0.001), but not for relative chlorophyll content (*p* = 0.142) (Table 1). Silicon addition and nitrogen level significantly increased the relative chlorophyll content of rice across all nitrogen levels (Figure 4a). Silicon addition significantly increased total mass, shoot mass, and root biomass under low and high nitrogen levels, but not under medium nitrogen level (Figure 4b–d). In general, relative chlorophyll content, total mass, shoot mass, and root mass of rice increased with increasing nitrogen level.

### 2.5. Effect of Silicon and Nitrogen Addition on Leaf Resistance Characteristics and Snail Feeding Metrics

**Figure 5:**
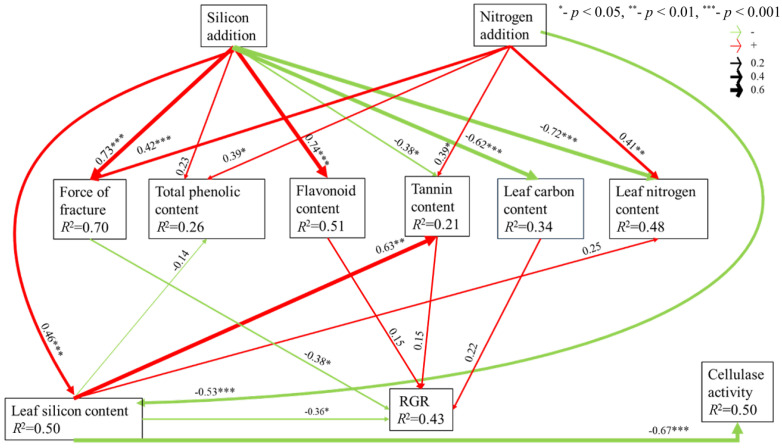
The effect of silicon addition and nitrogen addition on leaf resistance characteristics and leaf element content of rice, as well as the RGR and cellulase activity of apple snails, was analyzed using a structural equation model (SEM). Red and green arrows represent significant positive and negative pathways, respectively, and *** *p* < 0.001; ** *p* < 0.01; * *p* < 0.05. *R*^2^ represents the proportion of variance that is explained by each dependent variable in the model. The model’s Chi-square value was 29.605, with a *p*-value of 0.057. The AIC value was 797.9 and the GFI was 0.97.

Both silicon addition (standardized path coefficient *β* = 0.73, *p* < 0.001, Figure 5) and nitrogen addition (*β* = 0.42, *p* < 0.001, Figure 5) had significant positive effects on leaf fracture force. The combined effects of silicon addition, nitrogen addition, and leaf silicon content jointly accounted for 70% of the variance in force fracture. Nitrogen addition (standardized path coefficient *β* = 0.39, *p* < 0.05, Figure 5) also showed a significant positive effect on total phenolic content. Silicon addition had a positive effect on flavonoid content (*β* = 0.74, *p* < 0.001, Figure 5). On the whole, silicon addition, nitrogen addition, and leaf silicon content explained 51% of the variance in flavonoid content. Silicon addition showed a significant negative effect on tannin content (*β* = −0.38, *p* < 0.05, Figure 5), whereas nitrogen addition (*β* = 0.39, *p* < 0.05, Figure 5) and leaf silicon content (*β* = 0.63, *p* < 0.01, Figure 5) had positive effects. Silicon addition positively influenced leaf silicon content (*β* = 0.46, *p* < 0.001, Figure 5), while nitrogen addition had a negative effect (*β* = −0.53, *p* < 0.001, Figure 5). Silicon addition and nitrogen addition jointly accounted for 50% of the variance in leaf silicon content. Fracture force had a significant negative effect on the RGR of apple snails (*β* = −0.38, *p* < 0.05, Figure 5). Additionally, leaf silicon negatively affected both RGR (*β* = −0.36, *p* < 0.05, Figure 5) and cellulase activity (*β* = −0.67, *p* < 0.001, Figure 5) of apple snails.

## 3. Discussion

**Figure 6:**
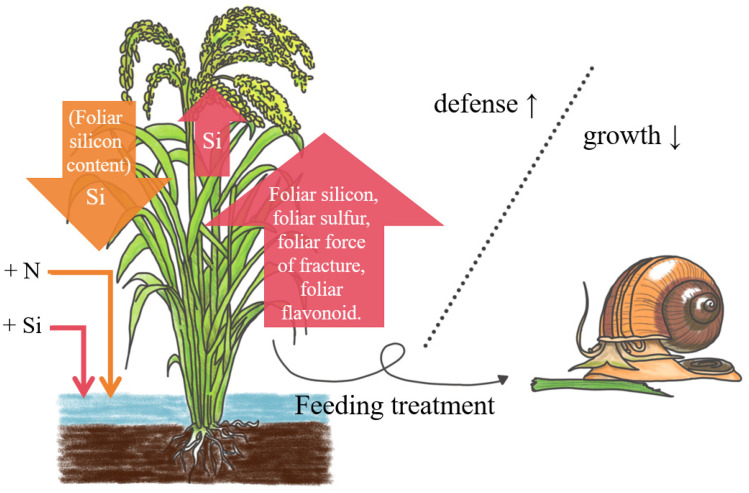
Conceptual diagram of how silicon application enhances rice resistance against apple snails. Silicon addition increases foliar silicon content, which promotes physical (force of fracture) and chemical (flavonoid, sulfur) defenses, ultimately suppressing snail growth. Nitrogen addition modulates plant growth and interacts with silicon, influencing overall resistance.

Silicon addition altered plant functional traits and secondary metabolite levels, which in turn influenced the ability of plants to defend against herbivores. The results obtained in this study indicate that the resistance of rice plants to invasive apple snails was affected by both silicon and nitrogen addition (Figure 6).

### 3.1. Effects of Silicon on Rice Growth in Nitrogen Treatments

Numerous studies have demonstrated that silicon plays an important role in plant responses to both biotic and abiotic stresses [21,22,23]. Nitrogen availability is also a major determinant of plant growth and crop productivity [42,43]. In our study, silicon application significantly increased rice biomass under both low- and high-N conditions, indicating that silicon promoted rice growth across contrasting N supplies. Our results also showed that high N supply significantly decreased leaf silicon content. This finding is consistent with Wu et al. [41], who reported that high N downregulated *OsLsi1* and *OsLsi2*, two transporters involved in Si uptake and transport, suggesting that N availability may influence Si acquisition in rice. In addition, the decrease in plant Si content under high N supply may also be associated with biomass dilution [44]. Leaf Si content tended to decrease with increasing shoot biomass, although this relationship was not statistically significant (Figure S1, *R*^2^ = 0.077, *p* = 0.596). de Tombeur et al. [44] similarly found that nitrogen addition reduced Si content in most wheat genotypes and that this reduction was strongly associated with increased biomass. Therefore, although biomass dilution was not statistically supported by our data, it remains a plausible mechanism contributing to the decrease in leaf Si content under high N supply. Silicon application also significantly increased plant sulfur content, suggesting that the effects of silicon extend beyond its interaction with N supply. Consistent with our findings, Barreto et al. [45] reported that silicon promoted sulfur accumulation under sulfur-deficient conditions.

Increasing nitrogen application can induce soil acidification, leading to asynchronous responses in micronutrient availability in both plants and soil. Sodium metasilicate has been shown to increase soil pH [46], thereby alleviating soil acidification and the associated asynchronous micronutrient responses induced by silicon addition in rice. Furthermore, high nitrogen levels reduce leaf silicon concentration in rice, while silicon application can mitigate the effect. However, under medium-to-high nitrogen conditions, silicon addition did not significantly affect plant biomass, although it improved the leaf C/N ratio in rice. The C/N ratio is commonly used as an indicator of plant growth and flowering potential, and a higher C/N ratio is generally associated with better nitrogen use efficiency [47]. The effect of silicon addition on rice biomass under medium nitrogen conditions was not significant, perhaps because the beneficial effects of silicon are more likely to manifest under suboptimal growth conditions such as nutrient limitation or nutrient imbalance, and are not significant under non-stress conditions [48]. This finding implies that silicon may enhance rice growth through two potential mechanisms. On the one hand, silicon addition increased the relative chlorophyll content of rice leaves across all nitrogen conditions, thereby improving photosynthetic rate. On the other hand, silicon addition increased the leaf sulfur content.

### 3.2. Effects of Silicon in Rice Resistance Under Nitrogen Treatments

To defend against herbivores, plants have evolved diverse mechanisms involving various chemical and physical traits [49]. Silicon typically contributes to plant defense against herbivores by forming mechanical barriers and is strongly associated with biochemical attributes [50]. Silicon is deposited in leaf hairs, trichomes, and spines, thereby influencing the physical traits of leaves [51]. In the present study, silicon addition significantly increased the fracture force of rice leaves. Previous research has shown that an increase in silicon content results in an increased force required for a needle to penetrate the leaf sheath [52]. Plant toughness can deter consumption by herbivores; force of fracture may provide an indication as to which plant might be more difficult for snails to consume [53,54]. Leaves with higher silicon content are more resistant to tearing; boosting Si levels in cereals has potential as a novel tool in crop protection against pest slugs and snails [55,56]. By covalently crosslinking with hemicelluloses, silicon addition enhances the mechanical properties of plant cells [57]. Furthermore, silicon has been demonstrated to activate the jasmonic acid signaling pathway, which is associated with plant defense against herbivores, thereby improving defense-related capabilities. Previous studies have reported that silicon can promote the production of defense-related enzymes, including polyphenol oxidase (PPO), superoxide dismutase (SOD), and peroxidase (POD) [58]. These enzymes play an essential role in plant defense by directly injuring herbivores or by promoting the accumulation of secondary metabolites, such as flavonoids and phenolics, which are harmful or toxic to herbivores [59].

Sulfur is an essential trace element for plant growth and development [60]. It is commonly found in proteins, peptides, metabolites, and enzymes [61]. Sulfur-containing metabolites and proteins play an indispensable role in plant heavy metal detoxification, antioxidant defense, and maintenance of cellular reducing conditions. Recent research has shown that silicon positively regulates the sulfur transporter gene *HvST1*, leading to increased SO_4_^2−^ uptake in *Hordeum vulgare* cv. Irina under combined stress conditions [62]. Another study demonstrated that silicon enhanced sulfur uptake and utilization in basil (*Ocimum basilicum*) [45]. This research showed that silicon improved sulfur accumulation in rice under sulfur-rich conditions. Silicon addition significantly increased the production of total phenolic and flavonoid, as well as leaf fracture force, across all nitrogen levels. Additionally, rice supplemented with silicon under high nitrogen levels exhibited a reduction in tannin content. Environmental factors, particularly temperature and soil nitrogen concentration, are known to significantly influence tannin concentration in plant leaves [63]. This study provides experimental evidence that silicon addition enhances the defense capacity of rice across different nitrogen levels.

### 3.3. Effects of Feeding Rice Leaves Experiencing Silicon and Nitrogen Addition on Apple Snails

Silicon addition reduced the growth of apple snails and decreased the consumption of rice leaves. Nitrogen acquisition is a critical determinant of herbivore growth, and high silicon content in leaves has been shown to reduce leaf digestibility, thereby limiting nitrogen acquisition in herbivores such as *Spodoptera exempta* [64]. The growth of apple snails is strongly influenced by plant nutrient availability, particularly nitrogen [65]. Nitrogen addition can lead to an increase in cellulose; applying a moderate amount of nitrogen can increase the plant’s resistance. But as the nitrogen content rises, the plant’s resistance decreases, making it more susceptible to herbivory [66]. Consistent with these findings, this study showed that silicon addition decreased leaf nitrogen content across all nitrogen levels. In addition, foods with a low C/N ratio can promote snail growth [67]. According to our findings, silicon addition resulted in a higher C/N ratio in rice leaves across all nitrogen levels. Silicon can also disrupt the integrity of epidermal cells in the herbivore gut, thereby impairing herbivore digestion [68]. Furthermore, silicon addition increases the activity and production of a variety of enzymes, such as proteinase and lipoxygenases [69]. The upregulation of defense-related genes leads to increased phytohormone activity and enhanced production of defense-related secondary metabolites, including phenolics, tannins, and flavonoids [70].

Phenolic compounds in green plants represent an important trait of plant defense against herbivores [71], and they can bind with proteins, leading to the damage of biological enzymes, thereby reducing both amino acid absorption and protein hydrolysis in herbivores [72]. Phenolics play a crucial role in limiting snail growth; consuming plant leaves with high phenolic content can inhibit snail development and may even cause mortality [65]. Tannins act as insect repellents by containing toxins that adversely affect their growth and survival [73], and they significantly reduce herbivore growth by limiting protein digestion efficiency [74]. Flavonoids serve as an effective means of plant defense against generalist herbivores and play important roles in host recognition and inhibition of herbivore feeding [75]. However, results from structural equation modeling (SEM) analysis indicated that flavones and tannins had no significant impact on RGR or cellulase activity of apple snails. The growth of apple snails was significantly influenced by total phenolic content. In this study, neither tannin nor flavonoid content significantly affected cellulase activity of apple snails. Furthermore, the feeding experiment was conducted for only one week, and the effects of tannin and flavonoid content on the RGR of apple snails have yet to be conclusively demonstrated. The results showed that silicon addition decreased the cellulase activity of apple snails. Cellulases are a group of hydrolytic enzymes that digest plant cell walls and decompose biological macromolecules into digestible, nutritive oligomers and monomers [76]. *P. canaliculata* has high cellulase activity; this activity affects how the golden apple snail digests the fiber in leaves [77]. Additionally, silicon addition increased phenolic and flavonoid content as well as leaf fracture force in rice across all nitrogen levels, leading to reduced leaf consumption and a negative effect on the growth of apple snails.

### 3.4. Implications for Control of Apple Snail Damage in Invaded Rice Agricultural Systems

Rice is a staple crop that provides food for nearly half of the world’s population [78]. Ensuring stable rice production is therefore crucial for global food security. However, biological invasions cause substantial damage to farmland ecosystems, leading to significant declines in food production [79,80]. In particular, the invasion of the apple snail in Southeast Asian countries has severely reduced rice yields. The invasive snail preferentially feeds on rice seedlings, as young plants are more suitable for its diet [81]. The snail typically feeds on the base of rice leaves before cutting through the entire plant, ultimately killing the seedling [82]. Therefore, controlling biological invasions is crucial for maintaining high rice yields and promoting sustainable rice agriculture systems. This study found that silicon can effectively mitigate the negative impacts of apple snail invasions in rice production by enhancing both plant growth and defense mechanisms. Silicon concentration plays a vital role in plant growth and development. Wongla et al. [83] found that under drought and salt stress, adding 1.5 mM and 2.0 mM of silicon increased the relative water content in rice, and 2.0 mM of silicon boosted the photosynthetic activity of rice. However, Significant differences exist in the silicon uptake capacity of plants, both across species and among genotypes [84]. Selecting rice varieties with a high capacity for silicon absorption is therefore a critical step [85]. In actual agricultural production, we suggest applying an appropriate amount of silicon fertilizer based on the soil and crops to boost rice’s resistance to apple snails. The role of silicon in the rice ecosystem, encompassing both its inputs and outputs, is essential and cannot be overlooked. Slag containing high levels of silicon has been widely used as a fertilizer for rice crops [86]. Rice stalks and other plant parts contain substantial amounts of silicon. Therefore, returning these residues to paddy fields is essential for maintaining the silicon cycle in rice agriculture systems [85]. Future research should focus on identifying suitable rice varieties and scientifically managing the silicon cycle in rice fields. Therefore, the approach will contribute significantly to the effective control of apple snail damage in invaded rice agricultural systems.

## 4. Materials and Methods

### 4.1. Experimental Design

#### 4.1.1. Experiment with N and Si Additions

From 15 March to 2 July 2022, preliminary plants were cultivated in the greenhouse of the Xiasha Campus of Hangzhou Normal University (30°32′ N, 120°40′ E), Hangzhou, Zhejiang, China. The greenhouse was maintained at an average temperature of 25 °C. Rice seeds (Jiahe 212A) obtained from the Chinese Academy of Agricultural Sciences were disinfected with alcohol, placed on wet filter paper for germination, and then transferred to seedling boxes filled with peat soil. Seedlings that had reached approximately 10 cm in height were transplanted into pots containing 2 L of agricultural field soil. These pots were placed in the greenhouse described above. The experiment followed a 3 × 2 factorial design, comprising three nitrogen levels (0.72, 1.44, and 4.76 mM N, for low, medium, and high nitrogen, respectively) combined with or without 1.5 Mm Si addition (as sodium silicate); each of six treatments was replicated 12 times, with a total of 72 pots [41]. In the soil solution, the concentration of silicon ranges from 0.01 to 2.0 mM [87]. According to Wu et al. [41], the silicon concentration used in the experiment was set at 1.5 mM. Sodium chloride was added to the non-silicon treatments at equivalent concentrations [40] to ensure the consistency of sodium ions. The soil was kept under a water layer during the experiment. Silicon and nitrogen were added weekly for two months to avoid the negative effects of a short treatment duration. All seedlings were harvested two months after planting, when they had developed six to eight leaves. From each treatment group, six seedlings were used to determine plant indicator measurements, and the remaining six were reserved for the snail feeding experiment.

#### 4.1.2. Snail Feeding Experiment

A snail-feeding experiment was conducted from 2 July to 8 July 2022. Apple snails were fed leaves from the harvested rice plants that had previously been subjected to the six treatments in the plant growth experiment. One month before the feeding experiment, apple snails of similar size were collected from a stream in Hangzhou, Zhejiang, China (30°32′ N, 120°1′ E), and were subsequently acclimated in the laboratory on a diet of lettuce. The lettuce supply was reduced one week before and completely withheld two days prior to the start of the feeding experiment.

The experiment was conducted in an incubator (PHCbi, Ehime, Japan) with an average temperature of 25 °C and humidity of 60%. At the beginning of the experiment, 36 healthy adult apple snails of similar size were selected. Each snail was carefully wiped dry to remove surface moisture and then weighed. The snails were randomly divided into six groups of six individuals each. Each snail was placed in a separate circular container, yielding a total of 36 containers. The circular containers were transparent cylindrical plastic boxes with a height of 10 cm and a diameter of 15 cm. Each group was then provided with six treated rice leaves to feed on for a week. The rice leaves provided were 5 cm in length and were replenished as needed based on daily consumption to ensure sufficient food for each snail. The rice leaves were replaced daily, and the consumed leaf was recorded. At the end of the feeding period, all snails were weighed again to assess weight gain. Subsequently, all apple snails were stored in a freezer at −80 °C for subsequent determination of cellulase activity.

### 4.2. Rice Sample Collection and Analysis

The relative chlorophyll content (SPAD) was measured using a portable chlorophyll fluorometer (SPAD-502Plus, Konica Minolta Inc., Tokyo, Japan) when the rice plants had reached the six-to-eight-leaf stage. For each plant, three fully expanded leaves were selected for measurement, and the mean value was calculated to represent the plant’s relative chlorophyll content. Half of the rice plants were then harvested, rinsed with water, and gently blotted dry with paper towels. Each plant was separated into aboveground and belowground parts, and the fresh weight of each part was immediately recorded. The fracture force of rice leaves was determined using an electronic universal material testing machine (Instron 3343, Boston, MA, USA). Rice leaves of similar physiological condition were fixed vertically to the testing machine and pulled from both ends at a constant speed until fracture. The maximum force at fracture was recorded, and overall leaf toughness was estimated based on leaf blade thickness, width, and the maximum fracture force [56]. Plant samples were dried in an oven at 60 °C until a constant weight was reached. For each treatment group, the total biomass of the six rice plants was measured using an electronic balance.

The dried rice leaves were ground using a mixer ball mill (MM400, Retsch, Düsseldorf, Germany). The ground leaf samples were analyzed for carbon (C), nitrogen (N), and sulfur (S) content using an element analyzer (vario PYRO cube; Elementar, Hanau, Germany) [88]. For flavonoid extraction, approximately 0.02 g of ground leaf powder was extracted with 60% ethanol, and the flavonoid content was determined using a spectrophotometer according to the sodium nitrite–aluminum nitrate colorimetric method as described by Lin et al. [89]. Tannin was extracted from 0.1 g of rice leaf powder using ultrapure water at 80 °C, and the tannin concentration was then determined according to Makkar [90] using the Folin–Ciocalteu colorimetric method with a spectrophotometer (Agilent Technologies Inc., Santa Clara, CA, USA). Total phenolics were extracted from approximately 0.1 g of rice leaf powder using 60% ethanol, and the total phenolic content in the extract was determined following the method described by Cheng et al. [91] using the Folin–Ciocalteu colorimetric method with a spectrophotometer. Plant silicon content was determined by digesting samples with sodium hydroxide solution at high temperature, followed by quantification using the molybdenum blue colorimetric method [92]. For each element or organic compound, six rice plants per treatment group were analyzed, and the experiment was conducted in triplicate.

### 4.3. Snail Sample Analysis

The apple snails were placed in an ultra-low temperature freezer and subsequently thawed in an ice water bath. The intestines and stomach were dissected out, and the tissues were homogenized on ice using a mortar and pestle. Cellulase was extracted from the snail intestinal and stomach tissues using a sodium citrate buffer containing protease inhibitors. Cellulase activity was measured using the 3,5-dinitrosalicylic acid method described by Kim et al. [93]. Relative growth rate (RGR) of the snails was defined according to Johnson et al. [40]:

$$\mathrm{RGR}(\mathrm{mg}\;{\mathrm{mg}}^{-1}\;{\mathrm{day}}^{-1})=\left(\frac{\mathrm{Final}\;\mathrm{mass}\;\mathrm{after}\;\mathrm{feeding}-\mathrm{Initial}\;\mathrm{mass}\;\mathrm{before}\;\mathrm{feeding}}{\mathrm{Initial}\;\mathrm{mass}\;\mathrm{before}\;\mathrm{feeding}\cdot \mathrm{days}}\right)$$

### 4.4. Data Analyses

Two-way ANOVA was used to test the effects of nitrogen concentration and silicon addition on plant biomass, tannin content, flavonoid content, phenolic content, silicon content, mechanical force, leaf loss, and cellulase activity. Multiple comparisons were conducted to explore the significant differences among treatment groups subjected to different combinations of silicon addition and nitrogen treatment. Duncan’s multiple range test was employed to pinpoint significant differences between treatments, with a significance threshold set at *p* ≤ 0.05. All statistical analyses were performed using SPSS 20.0.

A structural equation model (SEM) was constructed using linear regression to examine the effects of silicon and nitrogen addition on leaf resistance, leaf element content, and snail feeding metrics. This model assessed how the treatments influence plant growth and leaf resistance, as well as how leaf element content affected snail feeding behavior. Due to collinearity issues, several indicators were excluded during model construction, including all plant growth variables, leaf sulfur content, and leaf C/N ratio. To achieve a model with a nonsignificant chi-square test (*p* > 0.05), snail weight gain and leaf consumption were also excluded. The SEM was implemented using the lavaan package in R version 4.3.3 [94,95].

## 5. Conclusions

Silicon addition promoted the growth of rice but inhibited the growth of apple snails. Specifically, silicon addition increased the production of secondary metabolites in rice, leading to enhanced resistance. Silicon addition reduced nitrogen content while increasing phenolic levels and leaf sulfur content. Apple snails thrived on food that is high in nitrogen and low in phenolics. Nitrogen addition increased both leaf fracture force and phenolic content. However, nitrogen addition limited the silicon absorption of rice, indicating a clear trade-off between the uptake of nitrogen and silicon. On the other hand, nitrogen addition increased leaf nitrogen content, which may promote the growth of apple snails. This study suggests silicon application represents a promising, environmentally friendly approach to alleviating damage caused by the invasive apple snails. Furthermore, the judicious use of nitrogen fertilizer in rice fields is essential for the management of apple snails.

## Figures and Tables

**Table 1:** Two-way ANOVA on leaf resistance characteristics, snail feeding metrics, leaf element content, and plant growth responses to silicon addition and nitrogen levels.

	Silicon (Si)		Nitrogen (N)		Si × N	
Related Variables	F	*p*	F	*p*	F	*p*
***Leaf resistance characteristics***						
Total phenolic content	1.149	0.292	5.254	**0.011**	0.129	0.897
Tannin content	0.478	0.495	7.030	**0.003**	12.363	<**0.001**
Flavonoid content	37.217	<**0.001**	2.074	0.143	1.262	0.298
Force of fracture	47.416	<**0.001**	12.676	<**0.001**	0.793	0.462
***Snail feeding metrics***						
Leaf consumption	5.477	**0.026**	1.698	0.200	1.361	0.272
Relative growth rate	22.227	<**0.001**	3.114	0.059	2.742	0.081
Weight gain	13.637	**0.001**	2.664	0.086	2.907	0.070
Cellulase activity	19.946	<**0.001**	11.96	<**0.001**	2.708	0.083
***Leaf element content***						
Leaf carbon content	16.604	<**0.001**	0.153	0.895	0.949	0.398
Leaf nitrogen content	23.120	<**0.001**	4.482	**0.020**	0.192	0.826
Leaf sulfur content	25.508	<**0.001**	0.351	0.707	0.753	0.480
Leaf silicon content	23.823	<**0.001**	22.407	<**0.001**	6.150	**0.006**
Leaf C/N ratio	4.737	**0.038**	2.132	0.136	0.374	0.691
***Plant growth***						
Relative chlorophyll content (SPAD)	50.203	<**0.001**	56.252	<**0.001**	2.081	0.142
Total mass	94.289	<**0.001**	47.264	<**0.001**	25.937	<**0.001**
Shoot mass	60.645	<**0.001**	28.744	<**0.001**	16.007	<**0.001**
Root mass	625.700	<**0.001**	386.760	<**0.001**	201.200	<**0.001**

Note: Bold font indicates *p* < 0.05.

## Data Availability

The data presented in this study are available on request from the corresponding author. The data are not publicly available due to privacy and ethical restrictions.

## Supplementary Materials

The following supporting information can be downloaded at: https://www.mdpi.com/article/10.3390/plants15152357/s1, Figure S1. Linear regression between biomass and foliar silicon concentration. Data points represent treatment means (± SE). The regression equation, coefficient of determination (*R*^2^), and *p*-value are shown in the upper left corner. Different colors indicate different treatment groups. The shaded area represents the linear regression fit with 95% confidence intervals. No significant correlation was detected between biomass and foliar silicon concentration (*R*^2^ = 0.077, *p* = 0.596).

## Abbreviations

The following abbreviations are used in this manuscript:

SPAD	the relative chlorophyll content
C	carbon
N	nitrogen
S	sulfur
Si	silicon
SEM	structural equation model
RGR	relative growth rate
PPO	polyphenol oxidase
SOD	superoxide dismutase
POD	peroxidase
