# Supplementary material for: Effects of Si and N Addition on Oryza sativa and Its Invasive Grazer Apple Snails (Ampullariidae): Resistance Traits and Feeding Metrics
Source: Plants (Basel). 2026 Jul 30;15(15):2357. doi: 10.3390/plants15152357 (PMC13467741; doi:10.3390/plants15152357)
Supplement: Supplementary file 1 [file plants-15-02357-s001.zip › plants-4400241-supplementary.pdf]

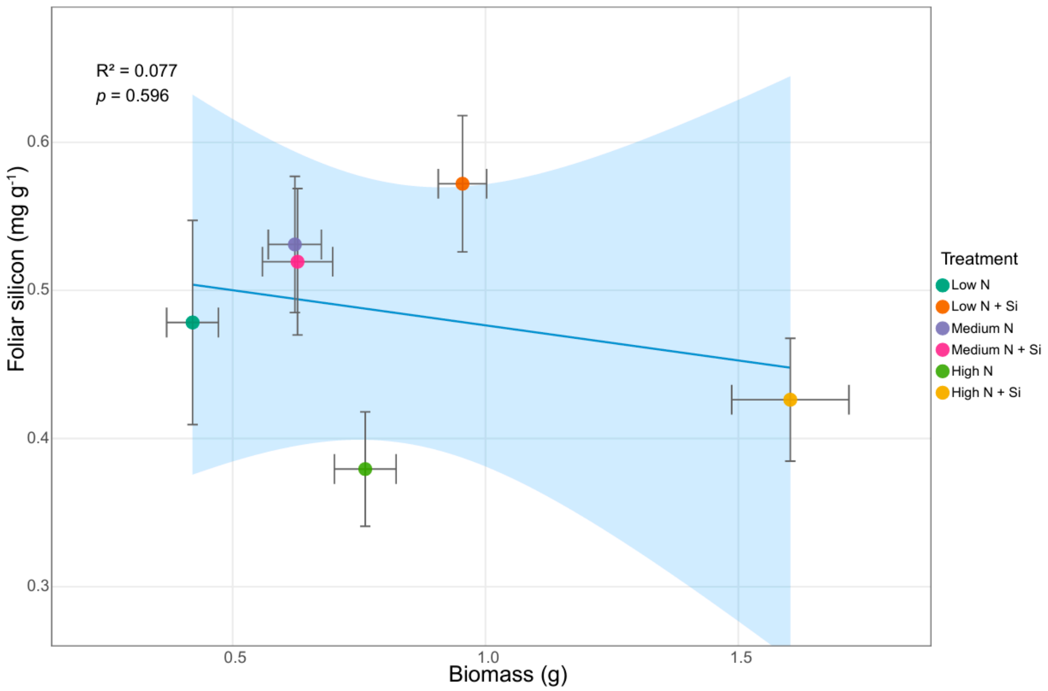

Figure S1. Linear regression between biomass and foliar silicon concentration. Data points represent treatment means (± SE). The regression equation, coefficient of determination (R^2), and p-value are shown in the upper left corner. Different colors indicate different treatment groups. The shaded area represents the linear regression fit with 95% confidence intervals. No significant correlation was detected between biomass and foliar silicon concentration ($R^2 = 0.077$, $p = 0.596$).
